# Establishment of a prognostic model of four genes in gastric cancer based on multiple data sets

**DOI:** 10.1002/cam4.3654

**Published:** 2021-05-02

**Authors:** Liqiang Zhou, Shi H. Li, You Wu, Lin Xin

**Affiliations:** ^1^ Department of General Surgery Nanchang University, The Second Affiliated Hospital of Nanchang University NanChang JiangXi 330006 China

**Keywords:** bioinformatics, biomarker, gastric cancer, nomogram, prognostic model

## Abstract

Gastric cancer (GC) is a kind of malignancy with a high mortality and recurrence. An effective prediction model based on ideal biomarkers to assess prognosis could benefit patients for optimization of treatment. Bioinformatics has played an increasingly important role in the study of cancer diseases. Therefore, this study started with bioinformatics to establish a reliable prognostic model of gastric cancer. The gene expression data and clinical data of GC tissues and normal tissues were obtained from the Gene Expression Omnibus (GEO), Genotype‐Tissue Expression (GTEx), and The Cancer Genome Atlas (TCGA) profile database. We finally identified a four gene signature and constructed a prognostic model. The results of internal and external validation showed that the model is highly reliable. In addition, we also constructed a nomogram based on the model, which was verified by a calibration curve to show its predicted accuracy. Comprehensive analysis indicated that the four genes in the model are related to the occurrence and development of tumors, perhaps they are potential targets for tumor treatment. Generally, this prognostic model can bring potential benefits to patients with gastric cancer.

## Graphical abstract

The model based on multi‐database bioinformatics analysis was used to predict the prognosis of gastric cancer.

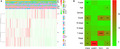

## 1 INTRODUCTION

Gastric cancer (GC) is the fifth most common cancer in the world, ranking third among all cancer deaths, and its incidence in East Asia, North America, and other places has increased significantly.^1^ Early gastric cancer was lack of specific symptoms, most of them develop into advanced gastric cancer with poor prognosis. The current treatments for gastric cancer are mainly surgery and chemotherapy, but the recurrence rate is high.^2^ Due to the poor prognosis of gastric cancer, a variety of new treatments and new prognosis‐related biomarkers have been proposed and applied, among which, new auxiliary chemotherapy and immunotherapy have been applied and shown good response in gastric cancer.^3^ However, how to choose the timing of the application of these new therapies is becoming a new challenge. Studies reported that the selection of drug for new complementary chemotherapy does not have a uniform standard and not completely effective in all types of gastric cancer, and that immunotherapy, especially for PD‐1/PDL‐1 inhibitors, did not show good suitability for patients with gastric cancer.^4^, ^5^ Therefore, how to choose treatment means needs to be combined with the patient's specific conditions such as tumor stage, size, grade, and other factors of comprehensive judgment. Individualized therapy can help patients with more suitable treatment by combining the actual situation of patients, and accurately predicting the prognosis of patients is one of the most important links in individualized treatment.^2^ Although various prognostic‐related biomarkers are recognized as effective in gastric cancer, it is still important to explore and identify new potential prognostic markers for gastric cancer diagnosis due to the complexity of the causes and mechanisms of development of gastric cancer.

Advances in tumor molecular biology have contribution to the development of predictive tools based on prognostic genes. Data from a variety of patients, including gene expression, survival, tumor stages, etc., are included in public databases, and researchers can perform bio‐informational analysis of these publicly available data to identify new prognostic‐related genes or summarize the underlying genetic characteristics of disease.^6^ A large amount of data can reduce the error caused by the analysis, and the conclusions of the analysis also have a wider applicability. Databases, such as TCGA, GEO, and GTEx, have been widely used in cancer research, which indicated that the analysis of a large number of data to determine tumor prognosis characteristics and prognostic markers is more beneficial.

In this study, we identified four genes associated with the prognosis model of potential gastric cancer by analyzing the difference expression genes (DEG) of normal tissue and stomach cancer tissue in the TCGA and GEO databases, and based on this, we established a prognostic model. The results of data set verification showed that the model is applicable and plays a great role in the prediction of gastric cancer.

## 2 MATERIALS AND METHODS

### 2.1 Acquisition of genetic and clinical data

Data files of FPKM expression in gastric adenocarcinoma (STAD) and corresponding normal tissues (375 tumor, 32 normal) were derived from TCGA (https://portal.gdc.cancer.gov). TPM value of normal tissues (359 normal) was derived from GTEx (https://gtexportal.org/home/). TCGA‐STAD clinical data were derived from cBioPortal^7^ database (www.cbioportal.org). Thirteen original files in GPL570 platform gene chip of gastric cancer tissue microarray (GSE66229, GSE54129, GSE13911,^8^ GSE19826,^9^ GSE79973,^10^ GSE51725,^11^ GSE15459,^12^ GSE51105,^13^ GSE35809,^14^ GSE57303,^15^ GSE34942,^16^ GSE22377,^17^ GSE38749) were obtained from GEO (https://www.ncbi.nlm.nih.gov/geo) (Table 1).

**Table 1:** GEO data sets included in this study

GEO data sets	Year	Country	Platform	Sample	Tumor (*n*)	Normal (*n*)
GSE66229	2015	USA	GPL570	GC/Normal	300	100
GSE54129	2017	China	GPL570	GC/ Normal	111	21
GSE13911	2008	Italy	GPL570	GC/ Normal	38	31
GSE19826	2010	China	GPL570	GC/ Normal	12	15
GSE79973	2016	China	GPL570	GC/ Normal	10	10
GSE51725	2013	Japan	GPL570	GC/ Normal	8	2
GSE15459	2009	Switzerland	GPL570	GC	200	0
GSE51105	2014	Australia	GPL570	GC	94	0
GSE35809	2012	Singapore	GPL570	GC	70	0
GSE57303	2014	China	GPL570	GC	70	0
GSE34942	2014	Singapore	GPL570	GC	56	0
GSE22377	2011	Germany	GPL570	GC	43	0
GSE38749	2012	Brazil	GPL570	GC	15	0

### 2.2 Data processing

The data downloaded from TCGA were converted into TPM value, and the “sva”^18^ package was used to combine with the TPM value from GTEx to remove batch effect. After removing the genes with mean less than 0.5, the “limma”^19^ package was used for difference analysis, set *P* < 0.05 and FDR (error detection rate) <0.05. The “affy”^20^ package was used to extract GPL570 platform chip‐expression data in GEO database. The “sva” package was used to combine with the abovementioned data and remove the batch effect. The “limma” package was used to analyze the difference, set *P* < 0.05 and FDR < 0.05. SangerBox (www.sangerbox.com mapping tool was used to make the Venn map; 651 DEGs were selected to be co‐regulated.

### 2.3 PPI network construction

We used the string database (https://string‐db.org/) to construct the DEGs gene protein–protein interaction (PPI) network. We set the minimum correlation value to 0.40 and removed unconnected points to exclude less correlated connections. The results were imported into Cytoscape^21^(3.7.2) to visualize PPI network interactions. The network analysis tool was used to analyze the degrees between various proteins, and the degrees were arranged from the inside out according to their sizes. The MCOD tool of Cytoscape was used to analyze and determine the key modules in PPI network, and the most representative top 3 modules were obtained for further analysis.

### 2.4 Gene enrichment analysis

We used Kyoto encyclopedia of Gene and genome database (KEGG)^22^ (https://www.genome.jp/kegg/) and the Gene Ontology database (GO)^23^ (http://geneontology.org/) enrichment analysis to investigate the functions of the genes in these key modules of gastric cancer. According to KEGG and the GO, the WebGestalt^24^ (www.webgestalt.org) tool was used for the enrichment of the gene in top 3 modules, set threshold *P* < 0.05 and FDR <0.05. The “GOplot” package^25^ was used to visualize the enrichment analysis results.

### 2.5 Construction of prognostic model

We used univariate Cox regression analysis to screen out genes related to the overall prognosis in TCGA‐STAD, and further used the "glmnet" R package to study the significant genes related to the prognosis of gastric cancer patients. We used the multivariate Cox regression analysis to analyze independent prognostic hub genes in gastric cancer and built a risk ratio model based on this. The linear grouping method was used to combine the expression level with coefficient of each gene, and the risk score formula was obtained as follows:

Risk score= β_1_ ∗ Exp_2_ + β1 ∗ Exp2+β_1_ ∗ Exp_2_ +…β_i_ ∗ Exp_i_ (Exp is the expression level of each prognostic gene and β is its regression coefficient).

Based on risk scores, patients in the data set were divided into two groups with high and low risk. KM survival analysis was used to show different survival rates among two groups, with logarithmic rank sum test to evaluate the performance. To explore the diagnostic ability of different genes at other levels of clinical prognostic parameters, risk and clinicopathological heat map was drawn to compare differences in age, gender, grade, TMN stage, AJCC stage, MSI status, and survival status. Univariate and multivariate Cox proportional hazards regression analyses were used to assess the risk levels of different factors. To verify the accuracy, we established the ROC curve of the prediction.

### 2.6 Verification of multigene prognostic signatures

Data sets with clinical data from the GPL570 platform were used as test sets. We used the test sets to prove the accuracy and applicability of multigene prognostic signatures in gastric cancer. Each patient's risk score was calculated by the coefficients of the four genes which could divide patients into high‐risk or low‐risk groups. The multigene prognosis signatures were verified by the KM survival curve. The logarithmic rank test and ROC analysis were used to assess the performance of KM survival curve.

### 2.7 Construction and Verification of the nomogram

To develop a quantitative prognostic approach, we constructed nomogram to predict the impact of each gene on 1‐ to 5‐year overall survival. The nomogram was constructed from prognostic factors screened by univariate and multivariate Cox regression analyses. Based on multivariate Cox analysis, point scales in the nomogram were used to assign values to individual variables. We used a horizontal line to determine the points of each variable and calculated the total points for each patient by adding up the points of all variables, normalizing the distribution from 0 to 100. Then we established the performance of the calibration curve to visual nomogram. We compared the predicted and observed results in the calibration curve. The best prediction occurred when the slope was close to 1.

### 2.8 Verification of the Hub gene expression

To verify the expression differences and levels of P3H2, UHRF1, THY1, and C5 in tumor tissues and normal tissues, three data sets in the Oncomine database (www.oncomine.org), including Cho Gastric (n = 90), Chen Gastric (n = 132), and Cui Gastric (n = 160) were used to verify the survival‐related hub genes.

### 2.9 Statistical analysis

Wilcoxon test was used to test the difference of DEG expression between tumor tissues and non‐tumor tissues in TCGA data. The eBayes function of R Documentation was used to analyze the data obtained in GPL570 platform gene chip. Kruskal‐Wallis test was used to compare the differences of DEG in all groups. Chi‐square test (X^2^) was used to evaluate DEG expression and clinicopathological parameters. Kaplan‐Meier analysis verified by log‐rank test was used to contrast different survival rates between the high‐ and low‐risk groups. Univariate and multivariate survival analyses were performed using Cox proportional hazard regression models. External validation was performed by the unpaired t‐test in GraphPad Prism Statistics software (7.0), set *P* < 0.05.

## 3 RESULTS

### 3.1 Acquisition of DEGs

**Figure 1:**
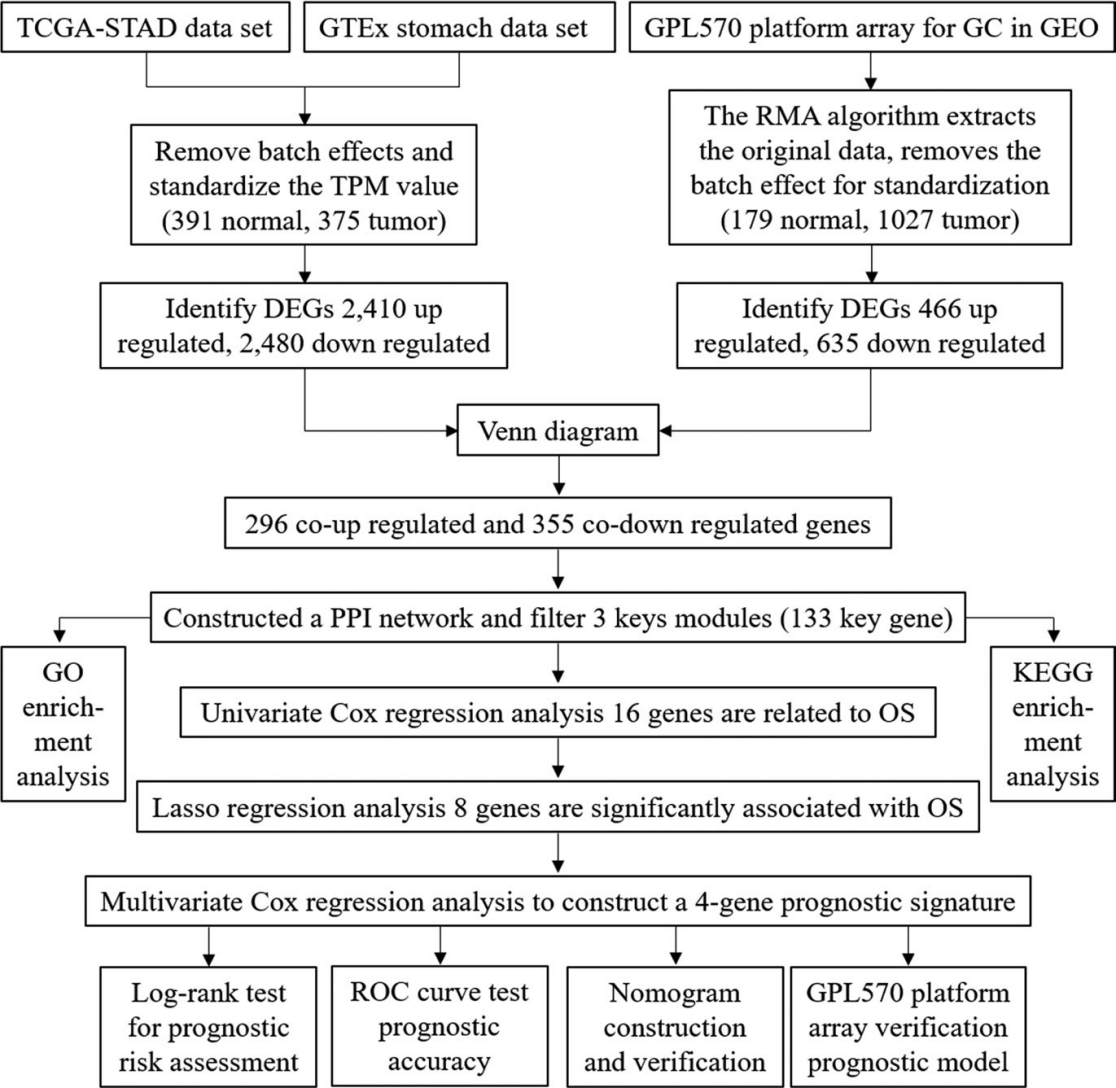
The workflow of entire research

**Figure 2:**
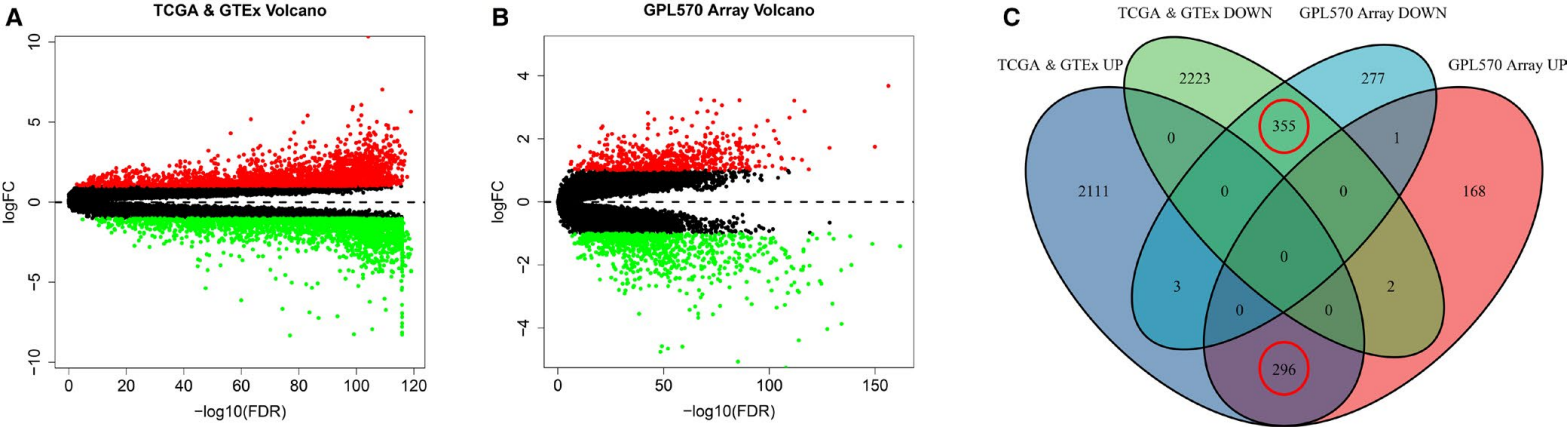
Regulated genes in normal and gastric cancer tissues. Each red dot represents an up‐regulated gene and each green dot represents a down‐regulated gene. (A) Volcano plot of differential expressed genes (DEGs) in TCGA & GTEx database. (B) Volcano plot of DEGs in GPL570 platform array. (C) Venn diagrams of the co‐regulated DEGs between tumor with normal tissues

The whole research process is shown in Figure 1. DEGs of tumor and non‐tumor tissues in TCGA database and GTEx database were analyzed by the “limma” package. The scatter plot showed the up‐regulation and down‐regulation of genes in gastric cancer and normal gastric tissue, and we got 2,410 up‐regulated and 2,480 down‐regulated DEGs (Figure 2A). The data obtained in the previous step and the data obtained from the GEO database were analyzed again by the “limma” package, 466 up‐regulated and 635 down‐regulated genes were identified (Figure 2B). Then, using Venn graph analysis, there were 296 co‐up‐regulated genes and 355 co‐down‐regulated genes in total (Figure 2C).

### 3.2 Construction of PPI network and DEGs enrichment analysis

**Figure 3:**
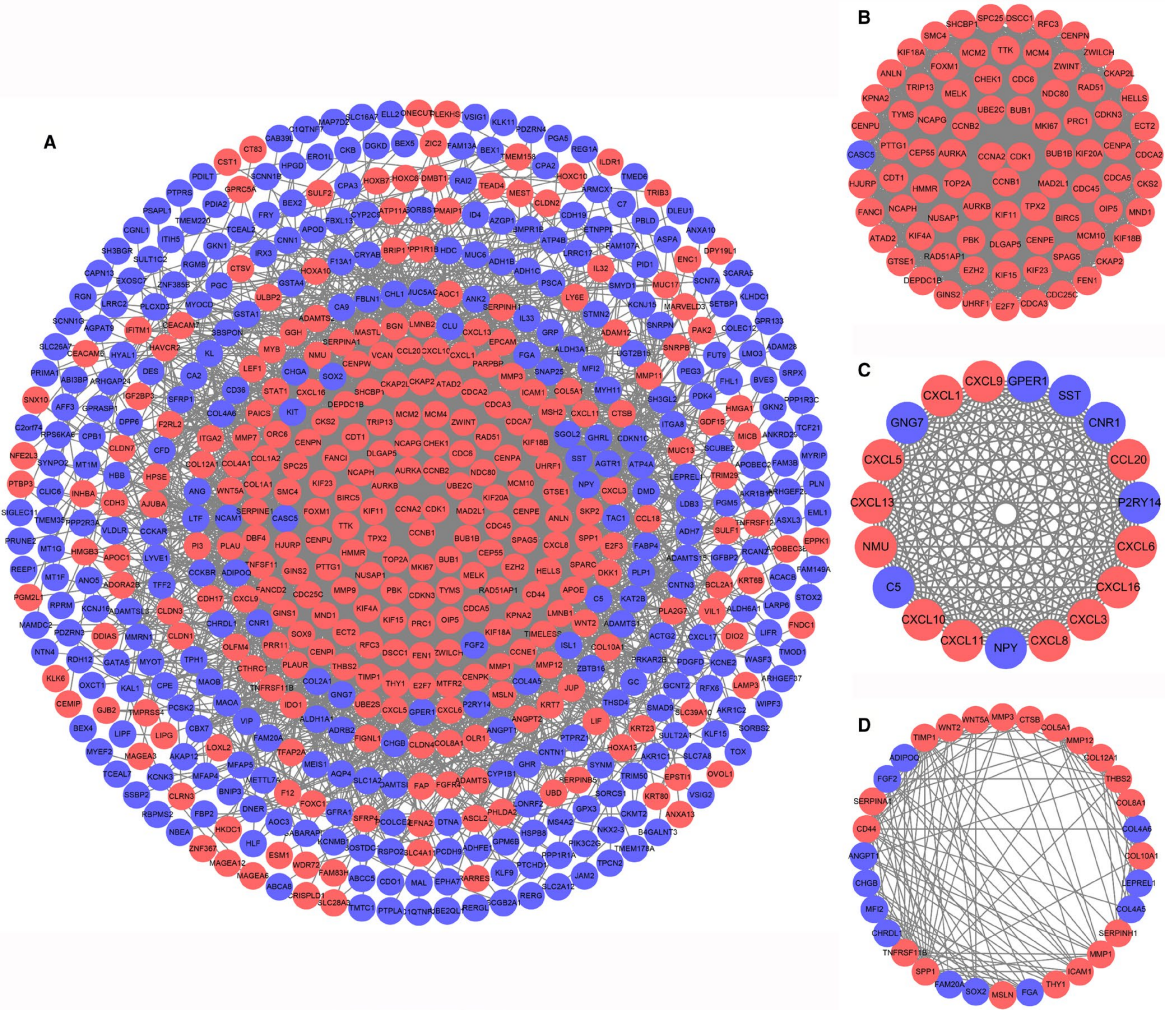
PPI network of all the co‐regulated DEGs and top 3 key modules. Up‐regulated genes are marked with red and down‐regulated genes are marked with blue. (A) PPI network of all the co‐regulated DEGs. (B) The key module 1. (C) The key module 2. (C) The key module 2

Based on the 651 DEGs obtained above, a PPI network with 591 nodes and 6,419 edges was constructed to identify the interactions among DEGs (Figure 3A). Node degree and intermediate degree were calculated by the MCC method to obtain hub node. MCODE analysis identified the three core modules with the highest scores in the PPI network (Figure 3B, C, D), which contained a total of 133 key DEGs, and these genes may play a core role in the PPI network.

**Figure 4:**
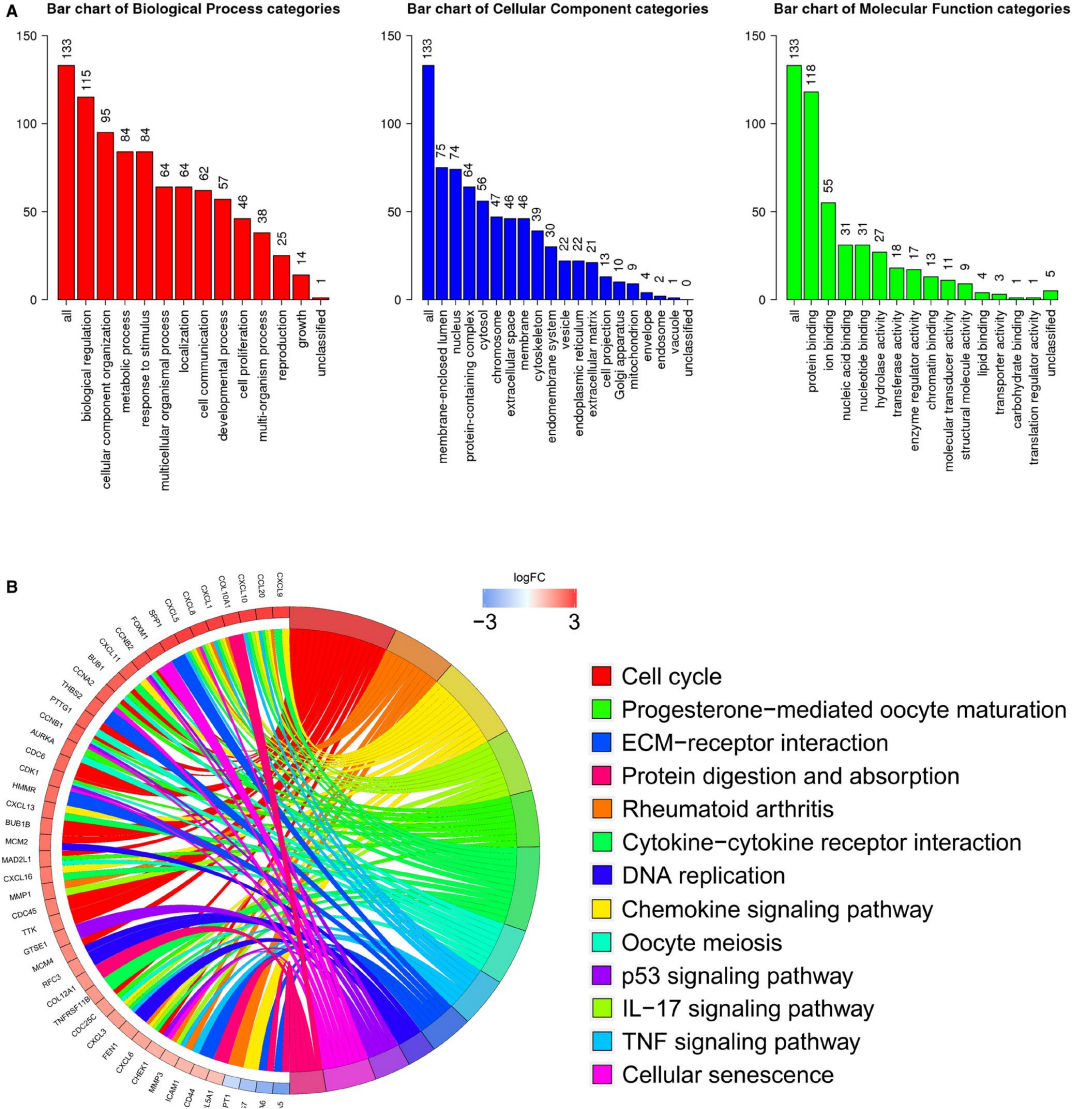
Gene enrichment analysis of 133 key DEGs in top 3 module of PPI network. (A) GO enrichment analysis. (B) KEGG enrichment analysis

Enrichment analysis by the GO and KEGG pathways was used to discover the function of key DEG. The key DEGs are expressed in cellular components (including the nucleus, cytoplasm, and membrane‐bound organelles), and are also significantly associated with cellular biological processes (including physiological regulation, metabolism, and stimulus response) and cellular molecular functions (such as binding of proteins, nucleic acids, and nucleotides) (Figure 4A). Figure 4B showed that some key DEGs were associated with cell cycle, DNA replication, and other processes. Some tumor‐related signaling pathways, such as p53, TNF, and IL‐17 pathways, were also connected with DEGs. These results indicated that the key DEGs are involved in various functions of tumor cells in varying degrees and may represent a key biological role in PPI networks.

### 3.3 Identification of survival‐related DEGs and establishment of four gene prognostic model

**Figure 5:**
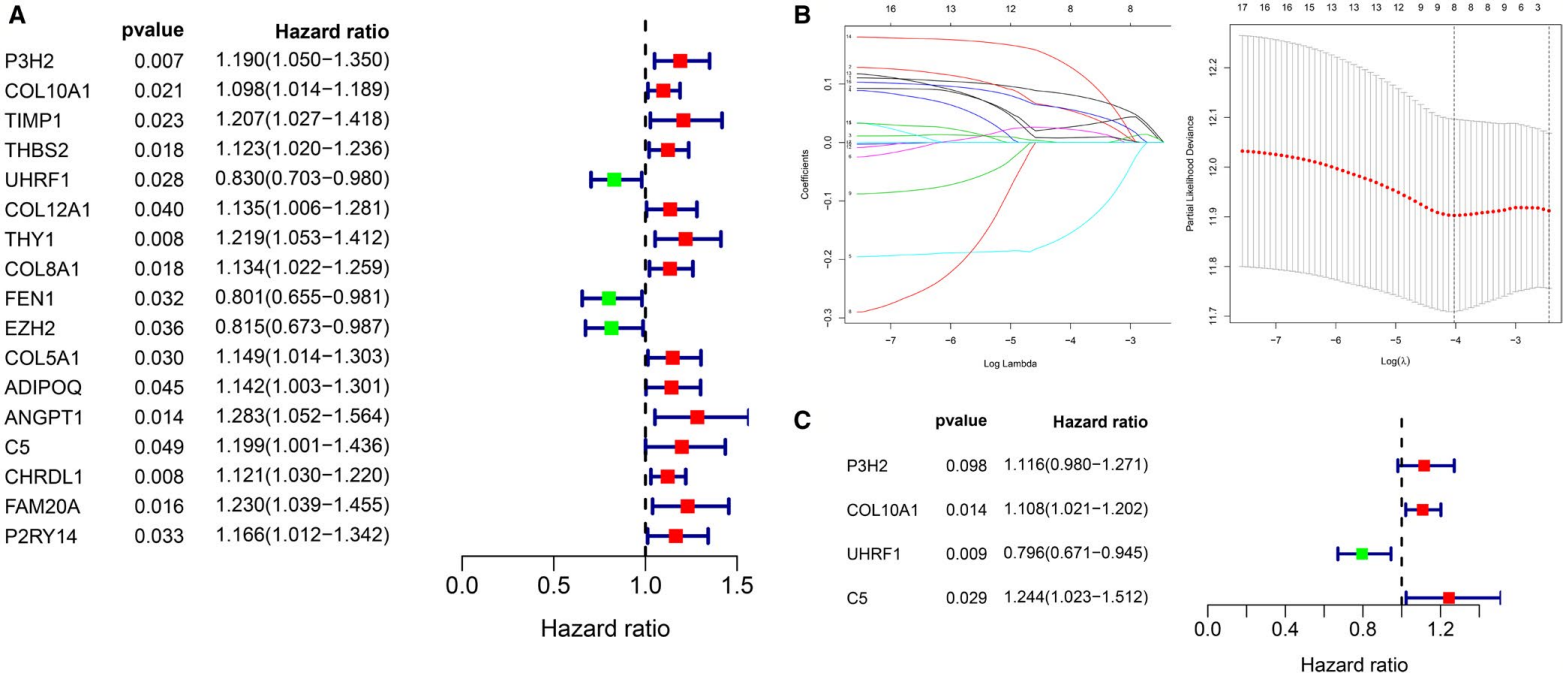
Identification of survival‐related DEGs. (A) A total of 16 genes associated with survival were identified by univariate Cox analysis. (B) Eight DEGs were further screened from 16 genes by lasso regression analysis and tenfold cross‐validation. (C) Multivariate Cox analysis finally screened out four optimal genes and constructed forest map

The results of univariate Cox analysis showed that there were 16 genes related to overall survival, among which up‐regulated genes were P3H2, COL10A1, TIMP1, THBS2, COL12A1, THY1, COL8A1, COL5A1, APIDOQ, ANGPT1, C5, CHRDL1, FAM20A, and P2RY14, and down‐regulated genes were UHRF1, FEN1, and EZH2 (Figure 5A). To further identify the DEGs significantly associated with GC prognosis, lasso regression analysis and tenfold cross‐validation were used to further screen for DEGs (Figure 5B). The best λ value (λ_min_ = 0.025) was obtained from the smallest local likelihood deviation, and eight genes were found that are significantly related to the prognosis. Multivariate Cox analysis directly identified four optimal prognostic gene models, namely P3H2, COL10A1, UHRF1, and C5 (Figure 5C). The risk scores of patients were calculated according to the scoring formula: Risk score = (P3H2 expression level * 0.109987) + (COL10A1 expression level *0.10246) + (UHRF1 expression level * −0.22772) + (C5 expression level * 0.218072).

**Figure 6:**
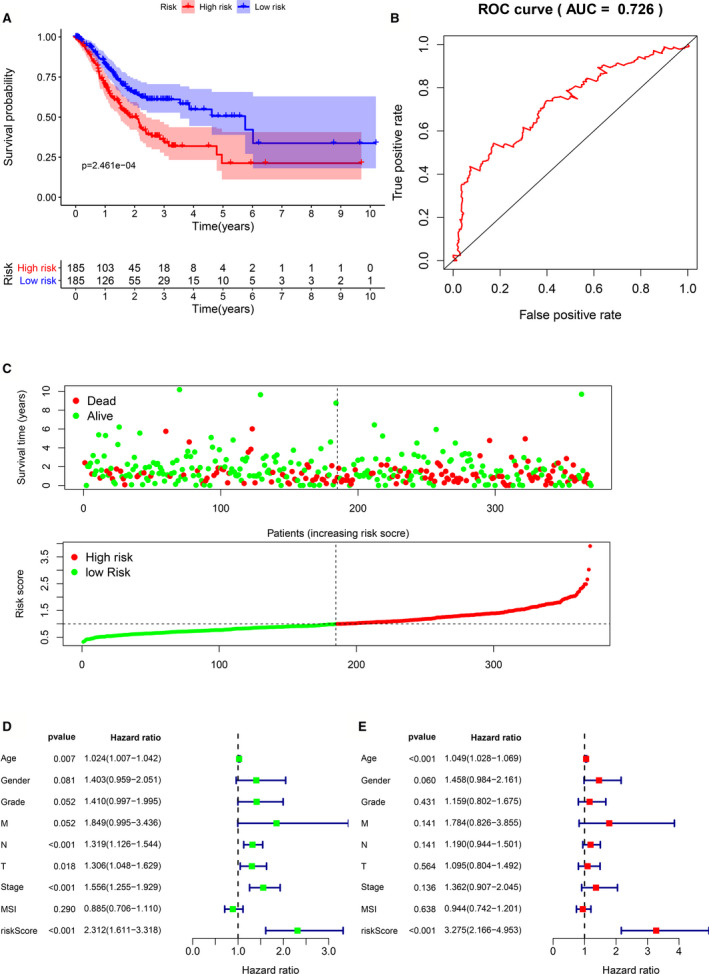
Predictive ability of four gene signatures. (A) Kaplan‐Meier survival curves for the high‐risk and low‐risk groups (B) ROC validation of the four gene signature, (AUC) =0.726. (C) The status distribution of patients’ survival and the heat map of hub gene expression. (D) Univariate Cox analyzes the relationship between clinicopathological characteristics and risk value and overall prognosis. (E) Multivariate Cox analyzes the relationship between clinicopathological characteristics and risk value and overall prognosis

Kaplan‐Meier survival curve evaluated the difference of survival between high‐risk and low‐risk patients in the risk model, and it was shown that the decrease in survival was more obviously in the high‐risk group than the increase in the low‐risk group over the time, mean prognosis of the high‐risk group is poorer than the low‐risk group (*P* = 2.461e‐04) (Figure 6A). Prognostic value of the Kaplan‐Meier survival curve was determined by the ROC curve. Area under the curve (AUC) of 0.726 indicated the median credibility of the Kaplan‐Meier survival curve (Figure 6B). The number of deaths in high‐risk group was higher than that in low‐risk group, and the number of patients with survival time of more than 5 years in high‐risk group was lower than that in low‐risk group. The heat map showed the risk with each prognostic gene. With the increase of risk score, the expression level of P3H2, COL10A1, and C5 in patients increased and UHRF1 was decreased (**Figure** **6C**). Univariate Cox analysis and multivariate Cox analysis were used to determine whether the risk value and other clinicopathological characteristics are independent prognostic factors. Univariate Cox regression analysis suggested that age [HR = 1.024, 95% CI (1.007–1.043), *P* = 0.007], N stage [HR=1.319, 95% CI (1.126–1.544), *P* < 0.001], T stage [HR = 1.306, 95%CI (1.046–1.629), *P* = 0.018], AJCC stage [HR = 1.556, 95% CI (1.255–1.929), *P* < 0.01], and risk score [HR = 2.312, 95% CI (1.611–3.318), *P* < 0.001] were related to the overall prognosis of gastric cancer patients (Figure 6D). Multivariate Cox regression analysis showed that the risk score [HR = 3.725, 95% CI (2.166–4.953), *P* < 0.001] was the best independent prognostic factor for gastric cancer patients compared with age [HR = 1.049, 95% CI (1.028–1.069), *P* < 0.001] (Figure 6E).

**Figure 7:**
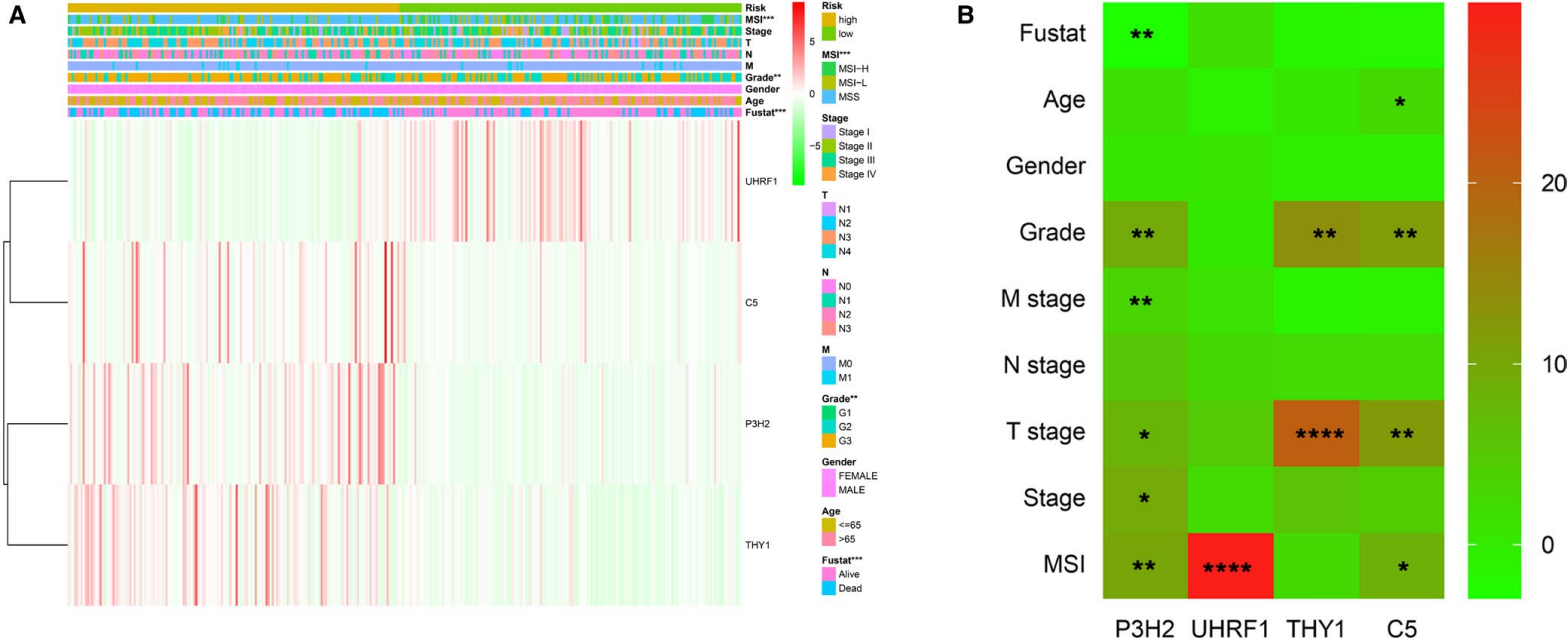
Correlation of risk value and four hub genes with clinical case characteristics. (A) Heat map of the correlation between risk value and clinical case characteristics. (B) Heat map of the correlation between the four hub genes and clinical case characteristics. *P < 0.05, **P < 0.01, ***P < 0.001, ****P < 0.0001

In addition, we further analyzed the risk score and the relationship between the four hub genes with clinical case characteristics in TCGA‐STAD. Figure 7A indicated that MSI status, Grade staging, and survival status are related to the level of risk. Among the four hub genes (Figure 7B), it is worth noting that the expression of P3H2 was significantly positively correlated with Grade, M, T, AJCC, and MSI status (*P* < 0.05). While the expression of HRF1 was only related to MSI status (*P* < 0.0001), the expression of THY1 and C5 was positively related to Grade and T stages (*P* < 0.05).

### 3.4 Verification of four gene prognostic signatures

**Figure 8:**
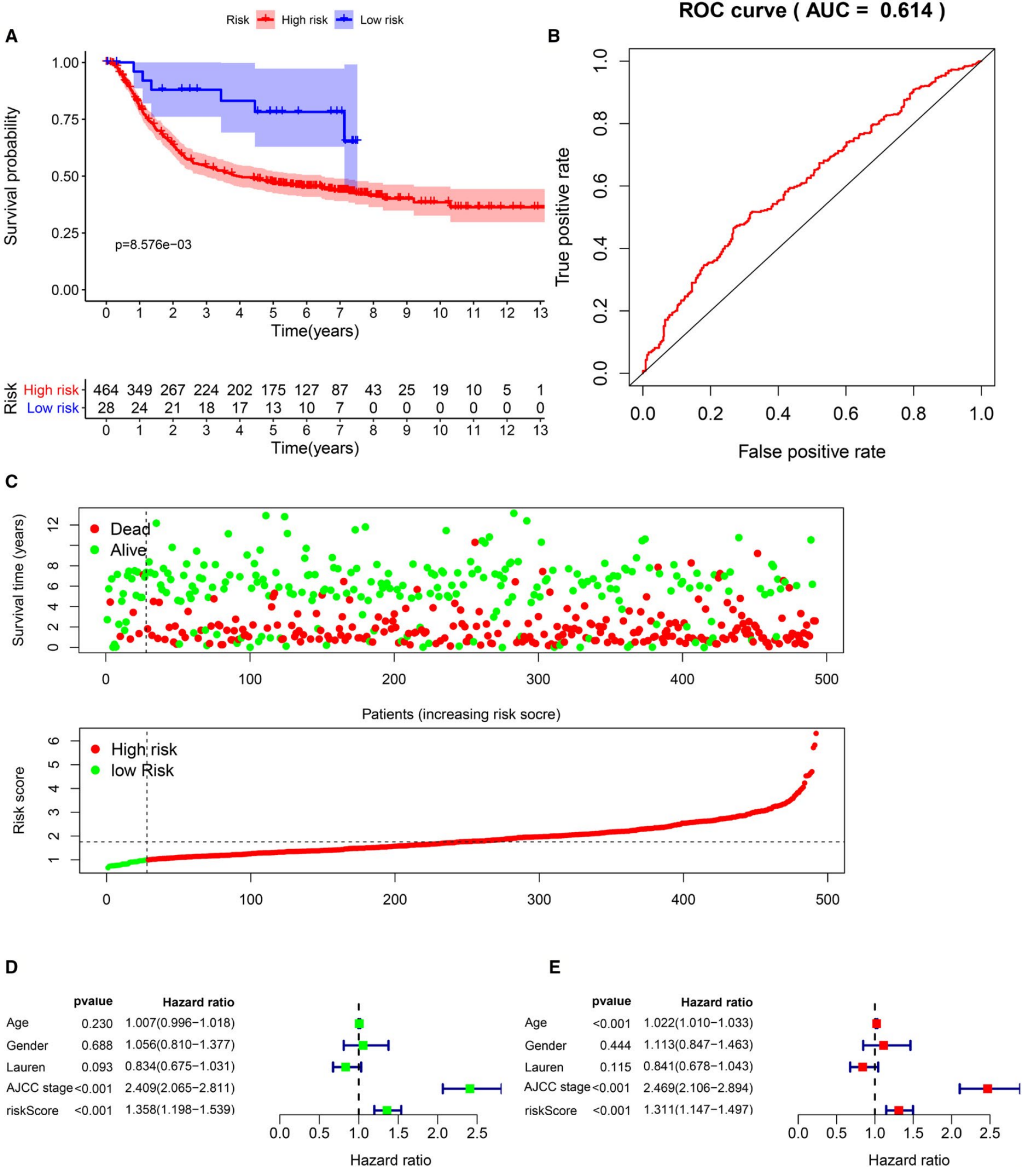
External verification of four gene signature. (A) Kaplan‐Meier survival curves for the high‐risk and low‐risk groups (B) ROC validation of the four gene signature, (AUC) =0.614. Patient survival status distribution and hub gene expression heat map. (D) Univariate Cox analyzes the relationship between clinicopathological characteristics and risk value and overall prognosis. (E) Multivariate Cox analyzes the relationship between clinicopathological characteristics and risk value and overall prognosis

Data from GPL570 platform arrays were used for external verification to test the applicability of the four gene prognostic signatures. In this data set, we analyzed a total of 493 gastric cancer patients. According to the previous risk score calculation formula, 464 patients were high risk and 28 patients were low risk. The results of Kaplan‐Meier survival curve showed a less optimistic prognosis in the high‐risk group than low‐risk group (*p* = 8.76e‐03), which is approximate to the results in the test set (Figure 8A). The AUC of ROC curve was 0.614, and showed that the prognostic value of four gene signature was high (Figure 8B). In addition, the heat map indicated that P3H2, COL10A1, and C5 was up‐regulated in the high‐risk group, while UHRF1 was low‐expressed in the high‐risk group, this was consistent with previous results (Figure 8C). Univariate Cox analysis (Figure 8D) and multivariate Cox analysis (Figure 8E) indicated that age [HR = 1.022, 95% CI (1.010–1.033), *P* < 0.001], AJCC stage [HR = 2.469, 95% CI (2.106–2.894), *P* < 0.001], and risk score [HR = 1.311, 95%CI (1.147–1.497), *P* < 0.001] were independent prognostic factors for gastric cancer patients, which was consistent with TCGA set.

### 3.5 Construction and verification of nomogram

**Figure 9:**
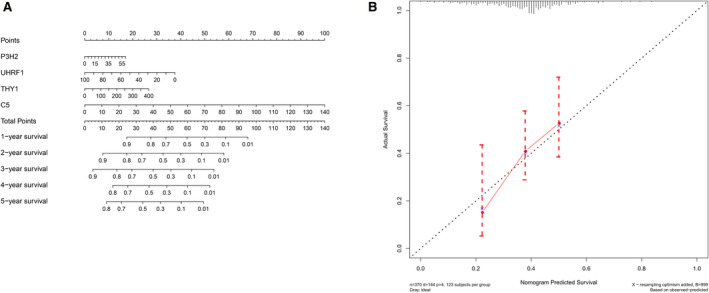
Establishment and validation of nomogram. (A) A prognostic nomogram was established for four hub gene expression level to predict 1–5 years of overall survival. (B) Calibration chart for nomogram. The dotted line represents the prediction results, and the red line represents the actual results. The high degree of coincidence between the two indicated that the prediction results are reliable

Based on 370 patients in TCGA‐STAD, we drew a nomogram composed of four hub gene expression levels. The corresponding assigned score was obtained by detecting the expression of the hub gene, and the total score was obtained by adding the individual gene scores, while the survival rate of gastric cancer patients can be predicted from 1 to 5 years (Figure 9A). Calibration curve was drawn to test the accuracy of the nomogram prediction. In the calibration chart, the predicted result (dotted line) was very close to the actual result (red line), means that the prediction of nomogram was of high quality (Figure 9B). In summary, four gene prognostic markers can accurately predict prognosis of GC patients.

### 3.6 External verification of four gene prognosis signature

**Figure 10:**
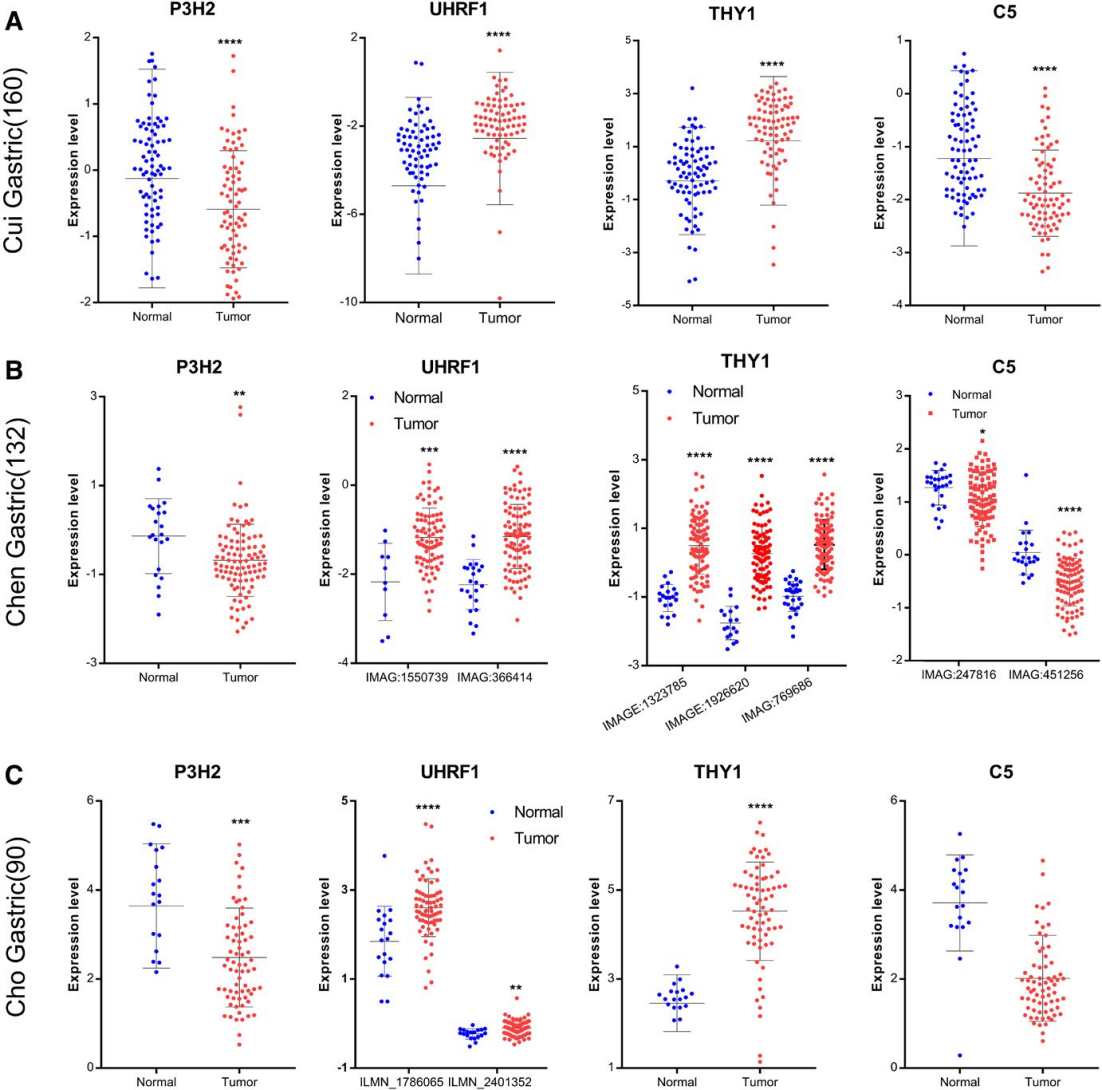
Oncomine database verified four hub gene expression. (A) Cho Gastric (n = 90) data sets. (B) Chen Gastric (n = 132) data sets. (C) Cui Gastric (n = 160) data sets. Blue represents normal, red represents GC, *P < 0.05, **P < 0.01, *** *P* < 0.001, ****P < 0.0001

Three data sets of Cho Gastric (n = 90) (Figure 10A), Chen Gastric (n = 132) (Figure 10B), and Cui Gastric (n = 160) (Figure 10C) were selected from the Oncomine database for external use to verify the expression level of central genes related to survival. Each gene was individually validated in three data sets. In general, the expression of P3H2, COL10A1, and C5 in tumor tissues was significantly increased than that in normal tissues, and the expression of UHRF1, considered as a tumor suppressor gene, was higher in normal tissues than in tumor tissues. The results of external validation were consistent with our previous results, suggested that the four gene signature can be used as a reliable prognostic model for gastric cancer.

## 4 DISCUSSION

Stomach cancer is one of the most common cancers in the world, and the low detection rate of early gastric cancer and the high recurrence rate of advanced stomach cancer together lead to a poor prognosis of stomach cancer. Individualized treatment of stomach cancer helps patients choose the most appropriate treatment among a variety of treatments, and accurate prediction of the patient's prognostication is critical for individualized treatment, with enables patients to choose more suitable therapeutic methods among molecular targeted, immunotherapy, and other new therapeutic methods.^26^ Molecular prognostic markers could be changed with tumor progression, and monitoring these markers can dynamically reflect the prognosis of patients, which may be further increased by combining AJCC stage, the most commonly used prognostic tool for gastric cancer.^27^ In addition, some molecular prognostic markers are involved in the development of tumors, they may be potential targets for tumor therapy and diagnostic indicators for early tumors.^28^ Molecular prognostic markers in different patients may be heterogeneous, so a group of molecular markers is better than a single molecular marker in terms of prognosis. In clinical practice, nomograms are widely used to evaluate multiple prognostic factors.^29^ Conventional AJCC staging combined with nomograms can provide more accurate predictions, which is undoubtedly beneficial to the individualized treatment of patients.

For a wide range of applicability, we analyzed and screened a large number of stomach cancer tissue and normal stomach tissue in the TCGA, GTEx, and GEO databases. One hundred and thirty‐three reliable key DEGs were identified through comprehensive analysis of multiple data sets. Gene enrichment analysis showed that DEGs were involved in many important biological processes such as protein synthesis, cell metabolism, and stimulus response. In addition, these DEGs are associated with several important tumor‐related pathways, such as p53, TNF, and IL‐17 pathways. Among them, p53 pathway is the most frequently activated pathway in cancer. Mutations in the tumor suppressor gene p53 are present in most of human cancers and have been shown to increase the ability of tumor invasion.^30^ The pro‐inflammatory role of IL‐17 pathway in human autoimmune diseases has been widely concerned. Recent studies have shown that IL‐17 pathway is also involved in the occurrence and development of tumors.^31^ TNF pathways are rich in functions and are involved in cell survival, apoptosis, and differentiation, hence their name due to their antitumor properties.^32^ To find the most representative survival‐related DEGs, we used univariate Cox analyses, lasso regression analyses, and multivariate Cox analyses for further analysis of these DEGs, resulting in p3H2, COL10A1, C5, and UHRF1 for a total of four genes. P3H2, COL10A1, and C5 were up‐regulated and associated with adverse survival, and UHRF1 was down‐regulated, indicating that this is a protective gene. Gastric cancer patients can be divided into high‐risk group and low‐risk group using these four gene signatures, and the survival rate of the high‐risk group is significantly lower than that of the low‐risk group. Similarly, Oncomine database was used to test the performance of prognosis models based on these four gene signatures, which showed that they performed well in predicting gastric cancer prognosis. All four genes were significantly related to survival and are involved in the biological processes of many important cells, suggesting their reliability as prognostic genes. P3H2 is a protein coding gene, and mutations in the gene are associated with severe non‐syndromic myopia with cataracts and vitreoretinal degeneration.^33^ In tumors, breast cancer may be associated with down‐regulation of the gene, but lung cancer studies have shown that up‐regulation of the gene is harmful to patients.^34^ The main role of COL10A1 gene is to promote cartilage ossification, and diseases associated with COL10A1 include metaphyseal chondrodysplasia, schmid type, and cartilage disease.^35^

Studies in gastric and colorectal cancer have shown that high expression of COL10A1 promotes epithelial‐mesenchymal transformation and tumor cell invasiveness and is associated with poor prognosis. It is worth noting that this study also used bio‐informational methods to reach this conclusion, further demonstrated that the COL10A1 gene played an important role in stomach cancer.^36^, ^37^ C5 gene plays a major role in the components of the coding complement system.^38^ The related pathways included immune response, lectin‐induced complement pathway, and GPCR signaling pathway. It has been reported that C5‐encoded PR proteolytic product C5 promotes tumor cell invasion, suggesting that up‐regulation of this gene in tumors promotes tumor progression.^39^ The protein UHRF1 encodes a hub protein that integrates epigenetic information. In the study of gastric cancer, the researchers found that the high expression of this gene would enhance the invasion and proliferation of tumor.^40^

P3H2 and C5 genes have not been reported in gastric cancer. However, it has been reported that these two genes are involved in tumor genesis and progression in other malignant tumors. In general, there are few reports on the role of these two genes in cancer, and further studies are needed to find out whether they play a role in the development of gastric cancer. The study showed that the abnormally high expression of COL10A1 promotes the proliferation, migration, and invasion of gastric cancer cells and is associated with poor tumor stage, which is consistent with our results.^41^ Studies on UHRF1 gene in gastric cancer showed that it promoted the proliferation of gastric cancer cells.^42^ Our study suggested that the down‐regulation of this gene leads to a poor prognosis, and the different observed results may be due to the unstable expression of this gene caused by the posttranslational modification.

Through the analysis of a large amount of data, the genes obtained in our study have a very broad applicability, which can bring some effect to the personalized treatment and accurate prediction of prognostics for gastric cancer patients. However,the potential mechanisms of the four hub genes that constitute the prognostic signature to regulate the occurrence and development of gastric cancer require further experimental studies. In addition, these genes need to be tested in larger clinical trials to further determine their suitability and accuracy.

## 5 CONCLUSION

In summary, our four gene expression prediction model based on multiple data sets is more economical and clinically feasible than whole‐gene sequencing. We also drew a nomogram of the prediction model, which can be used to evaluate the prognosis of different patients individually by detecting the expression of genes, which is undoubtedly more beneficial to the selection of effective treatment methods. In addition, the DEG was obtained from multiple data sets and verified, with high reliability, and may also be a potential target for anti‐gastric cancer.

## 6 Ethics Statement

All clinical data involved in this study were obtained from open databases and therefore met ethical review standards.

## CONFLICT OF INTEREST

The author claims no conflict of interest.

## AUTHOR CONTRIBUTIONS

Liqiang Zhou and Shihao Li: Conception, design, data analysis, and manuscript writing, You Wu: Data collection, Lin Xin: Guide, supervise, and review.

## Data Availability

All the data come from public databases.
